# Developmental Impairments of Synaptic Refinement in the Thalamus of a Mouse Model of Fragile X Syndrome

**DOI:** 10.1007/s12264-023-01142-6

**Published:** 2023-11-28

**Authors:** Xiaotong Wu, Yali Liu, Xiaomeng Wang, Lu Zheng, Libiao Pan, Hao Wang

**Affiliations:** 1 Department of Neurosurgery of Second Affiliated Hospital and School of Brain Science and Brain Medicine, Key Laboratory for Biomedical Engineering of Education Ministry, Zhejiang University School of Medicine, Hangzhou, 310058 China; 2 Nanhu Brain-computer Interface Institute, Hangzhou, 311100 China; 3 NHC and CAMS Key Laboratory of Medical Neurobiology, MOE Frontier Science Center for Brain Research and Brain Machine Integration, Key Laboratory of Precise Treatment and Clinical Translational Research of Neurological Diseases, School of Brain Science and Brain Medicine, Zhejiang University, Hangzhou, 310058 China; 4 Lingang Laboratory, Shanghai, 200031 China

**Keywords:** Fragile X syndrome, Synaptic refinement, VPm, Sensory over-reactivity

## Abstract

While somatosensory over-reactivity is a common feature of autism spectrum disorders such as fragile X syndrome (FXS), the thalamic mechanisms underlying this remain unclear. Here, we found that the developmental elimination of synapses formed between the principal nucleus of V (PrV) and the ventral posterior medial nucleus (VPm) of the somatosensory system was delayed in fragile X mental retardation 1 gene knockout (*Fmr1* KO) mice, while the developmental strengthening of these synapses was disrupted. Immunohistochemistry showed excessive VGluT2 puncta in mutants at P12–13, but not at P7–8 or P15–16, confirming a delay in somatic pruning of PrV-VPm synapses. Impaired synaptic function was associated with a reduction in the frequency of quantal AMPA events, as well as developmental deficits in presynaptic vesicle size and density. Our results uncovered the developmental impairment of thalamic relay synapses in *Fmr1* KO mice and suggest that a thalamic contribution to the somatosensory over-reactivity in FXS should be considered.

## Introduction

Human fragile X syndrome (FXS) is caused by the loss of fragile X mental retardation protein (FMRP). It is characterized by intellectual disability and is accompanied by features of autism spectrum disorders (ASDs) including problems with social interaction and altered sensory processing [1–4]. *Fmr1* knockout (*Fmr1* KO) mice are commonly used as a rodent model to study FXS [5]. They display many behavioral abnormalities similar to those found in FXS patients such as impairment in learning and memory [6, 7], deficits of social behavior [8–11], and abnormalities in sensory processing [12, 13]. Among those symptoms, somatosensory over-reactivity has recently been receiving closer attention linked to the recognition that irregularities in touch and tactile perception may be associated with broad levels of social dysfunction in ASDs. In the somatosensory cortex of *Fmr1* KO mice, deletion of FMRP has been shown to cause abnormal development of dendritic spines, exhibiting higher dendritic spine density and increased numbers of elongated immature dendritic structures compared to WT mice [14, 15]. Other studies using various ASD mouse models harboring mutations in the *Mecp2, Gabrb3, Shank3*, and *Fmr1* genes exhibit hypersensitivity to touch stimuli [16]. Especially, deletion of *Mecp2*, *Gabrb3,* or *Shank3* during development, but not in adulthood, in peripheral somatosensory neurons causes altered tactile sensitivity and social interaction deficits [17]. Such studies have revealed the cortical and peripheral mechanisms underlying somatosensory over-reactivity. However, considering that tactile information is transmitted to the cortex *via* the thalamus, we considered it of great interest to explore any contribution of thalamic synapses to the somatosensory over-reactivity in *Fmr1* KO mice.

Rodents largely rely on their vibrissal system to receive tactile information and explore their environment. Tactile information from the large rodent whiskers is relayed from the snout to the neocortex primarily through the lemniscal pathway, which involves the principal nucleus of V (PrV) in the brainstem and the ventral posterior medial nucleus (VPm) in the thalamus, but also *via* the paralemniscal pathway [18]. As glutamatergic synapses are formed by the projection of the PrV to VPm neurons, synapse refinement, the process of eliminating unwanted and consolidating desired synapses, plays a crucial role in the early development of the somatosensory system [19, 20]. These processes are vital for the formation of precise somatosensory circuits and lay an important foundation for the processing of tactile information. Previous studies have uncovered that VPm neurons first receive extensive innervation from the PrV at the postnatal day 7 (P7) developmental stage. Later, at P12–13, some of the redundant PrV inputs are eliminated causing the majority of VPm neurons to receive 2–3 PrV inputs. Then, by around P15–16, most of the VPm neurons simply receive a single PrV input [19]. Meanwhile, the remaining synapses become mature and strengthened through the incorporation of additional AMPARs (α-amino-3-hydroxy-5-methyl-4-isoxazolepropionic acid receptors) [21]. It is important to note that P12–13 is also the critical period for experience-dependent plasticity at the whisker sensory relay synapse in the VPm [20]. Despite synapse elimination and the remaining synapse strengthening invariable occurring concurrently, these two aspects of developmental synapse refinement are mechanistically distinguishable and, to a certain extent, independent. For example, the elimination of redundant inputs can take place normally, even without synapse strengthening [21]. Therefore, the VPm relay synapses provide an ideal model in which to investigate the distinct developmental processes of synapse elimination and synapse strengthening separately in *Fmr1* KO mice. Since the phenotype of somatosensory over-reactivity has an early onset, we focused on investigating the refinement of the VPm relay synapse during early development. This study may also shed light on the understanding of thalamic mechanisms for somatosensory over-reactivity in ASDs.

## Methods and Materials

### Animals

All procedures were in accordance with the NIH *Guide for the Care and Use of Laboratory Animals* and were approved by the Zhejiang University Animal Experimentation Committee (Protocol AIRB-2021-948). *Fmr1*^tm1Cgr^ transgenic male mice (RRID: IMSR_JAX:002700) were used and maintained by heterozygous mating. Mice were housed under a 12h light/dark cycle and provided food and water *ad libitum*. Heterozygous (*Fmr1*^+/−^) female mice and wild-type (WT) male (*Fmr1*^+/*y*^) mice were used for breeding to generate male hemizygous full-mutant mice (*Fmr1*^−/*y*^) and WT littermate controls (*Fmr1*^+/*y*^). Only male mice aged P7 to P16 were then used in experiments.

Genotypes were determined by PCR using the following three primers: 5′-CAC-GAG-ACT-AGT-GAG-ACG-TG-3′ (mutant forward), 5′-TGT-GAT-AGA-ATA-TGC-AGC-ATG-TGA-3′ (WT forward), and 5′-CTT-CTG-GCA-CCT-CCA-GCT-T-3′ (common), which amplified 131 and 400 bp fragments from the WT and mutated alleles, respectively.

### Slice Preparation

Brain slices were prepared as previously described [21]. Mice were anesthetized with sodium pentobarbital and then killed by decapitation. The brain was removed rapidly and immersed in ice-cold oxygenated slicing solution containing (in mmol/L): 110 choline chloride, 7 MgCl_2_·6H_2_O, 2.5 KCl, 0.5 CaCl_2_·2H_2_O, 1.3 NaH_2_PO_4_, 25 NaHCO_3_, and 20 D-glucose, saturated with 95% O_2_ and 5% CO_2_. Sagittal slices 300 μm thick were cut on a vibratome (Leica VT1200S, Weztlar, Germany). Slices were allowed to recover for 30 min at 32 °C and then at room temperature in artificial cerebrospinal fluid (aCSF) containing (in mmol/L): 125 NaCl, 2.5 KCl, 2 CaCl_2_·2H_2_O, 1.3 MgCl_2_·6H_2_O, 1.3 NaH_2_PO_4_, 25 NaHCO_3_, and 10 D-glucose. Oxygen was continuously supplied during recovery and recording. To block inhibitory synaptic transmission, picrotoxin (100 μmol/L) was added to the bath.

### Patch-Clamp Recording

Whole-cell patch-clamp recordings were made from the soma of VPm neurons at room temperature using a Multiclamp 700B amplifier and Digidata 1440A with pCLAMP 10.4 software (Molecular Devices, Sunnyvale, USA). Picrotoxin (100 µmol/L) was added to the bath to block GABAergic transmission. For voltage-clamp recording, the pipette was filled with an internal solution containing (in mmol/L): 110 Cs methane sulfonate, 20 TEA-Cl, 15 CsCl, 4 ATP-Mg, 0.3 GTP-Na, 0.5 EGTA, 10 HEPES, 4 QX-314, and 1 spermine (pH 7.2, 290–300 mOsm with sucrose). To record evoked quantal events, strontium (Sr^2+^) was applied to replace Ca^2+^ in the ACSF, which reduces the peak amplitude of EPSCs at −70 mV and causes a large number of asynchronous miniature EPSCs (Sr-EPSCs). Patch electrodes had a resistance of 2–4 MΩ. The series resistance (Rs) was usually 8–18 MΩ with no compensation. When the Rs had changed by >20%, the data were discarded. Signals were filtered at 2 kHz and digitized at 10 kHz.

Data of evoked quantal events were initially processed using Clampfit (Molecular Devices). The amplitude and frequency of Sr-EPSCs were analyzed using MiniAnalysis software (Synaptosoft, Decatur, USA) with manual *post hoc* verification.

To determine the number of inputs to each VPm neuron, we recorded evoked AMPAR-/NMDAR- EPSCs from the same cells at holding potentials of −70 /+40 mV over a wide range of stimulus intensity. A concentric electrode (World Precision Instruments, Sarasota, USA) was placed on the medial lemniscus, and stimuli (usually between 0.02 and 1.0 mA, 100 µs) were delivered at 0.1 Hz *via* a Master-8 stimulator (A.M.P.I., Jerusalem, Israel). We first used paired-pulse stimulation with an interval of 100 ms to distinguish lemniscal synaptic responses from corticothalamic responses. We then searched for step numbers roughly by increments of 50–100 µA and used small increments of 1–10 µA near each transition point to verify that this was indeed a single step. Finally, we used a stronger stimulus of at least twice the intensity that was evoked in the previous step. We carried out two or more trials at each stimulus intensity.

### Immunostaining and Imaging

Animals were anesthetized with sodium pentobarbital, and perfused with saline followed by 4% paraformaldehyde (PFA) in 0.1 mol/L phosphate buffer (PBS). Brains were removed, post-fixed for 5–6 h in 4% PFA, and then transferred to 30% sucrose and kept at 4 °C for 2 days. Sagittal sections were cut at 40 µm on a microtome (Cryostar NX50, Thermo, Waltham, USA). After washing three times with 0.01M PBS, rinsing with frozen methanol (10 min at -20 °C), and blocking with 10% bovine serum albumin (BSA) for 1 h at room temperature, the sections were incubated with primary antibodies as follows: anti-VGluT2 (guinea-pig polyclonal, 1:600, Millipore, Billerica, USA, Cat# AB2251-I, RRID: AB_2665454) and anti-NeuN (rabbit monoclonal, 1:500; Millipore Cat# MABN140, RRID: AB_2571567) at 4 °C for 12–24 h. After rinsing, secondary fluorophore-conjugated antibodies (Alexa Fluor 488, donkey anti-rabbit, 1:1000, Thermo Fisher Scientific Cat# A-21206, RRID: AB_2535792; Alexa Fluor Cy3, donkey anti-guinea pig, 1:1000, Jackson, West Grove, USA, Cat# 706-165-148, RRID: AB_2340460) were applied for 2 h at room temperature. The antibodies were diluted in PBS containing 5% BSA. Images were captured using a 60× objective on an Olympus FV-1000 or FV-1200 inverted confocal microscope. 10–12 consecutive images at 0.5 mm intervals were captured in Z-stacks.

### Quantification of VGluT2

Images were analyzed blindly using ImageJ (NIH, Bethesda, USA, version 1.51). Several VPm neurons were randomly selected from each slice. By using the Cell Counter plugin, the VGluT2 puncta on the soma were manually counted. The number of puncta in contact with the NeuN was determined as the puncta/soma. To quantify VGluT2 puncta/neuron, the Analyze Particles command of ImageJ was used to analyze the density of VGluT2 puncta in each image. The number of thalamic VPm neurons was then counted manually by the Cell Counter plugin. The puncta/neuron was calculated by dividing the total number of puncta by the total number of neurons.

### STEM-ET

The VPm of *Fmr1*^*-/y*^ mice and WT littermates (P7–8, P12–13, and P15–16) was dissected, trimmed into ~1 mm^3^ pieces, and fixed overnight with 2.5% glutaraldehyde in PBS buffer. After rinsing in 0.1M PBS (10 min, 3 times), the specimens were post-fixed with 1% OsO_4_ for 1 h, stained with 2% uranyl acetate for 30 min, dehydrated by ethyl alcohol and acetone, and then embedded. An ultrathin microtome (UC7, Leica, Germany) was used to cut 400 nm sections for electron tomography. For the 2D imaging of synaptic vesicles, electron micrographs were acquired using a Tecnai G2 Spirit 120 kV (Thermo FEI). The 2D images were then analyzed using ImageJ software (NIH, version 1.51).

### Statistical Analysis

All data are presented as the mean ± SEM. Statistical analysis was applied with GraphPad Prism 6 (GraphPad Software, La Jolla, CA). Student’s t-tests or Mann-Whitney tests were used as indicated for differences between the two groups. Frequency distributions were analyzed using a χ^2^ test. For all statistical analyses, significance was set at *P <0.05, **P <0.01, ***P <0.001, ****P <0.0001.

## Results

### Developmental Synapse Elimination is Delayed at the VPm Relay Synapses in *Fmr1* KO Mice

**Fig. 1:**
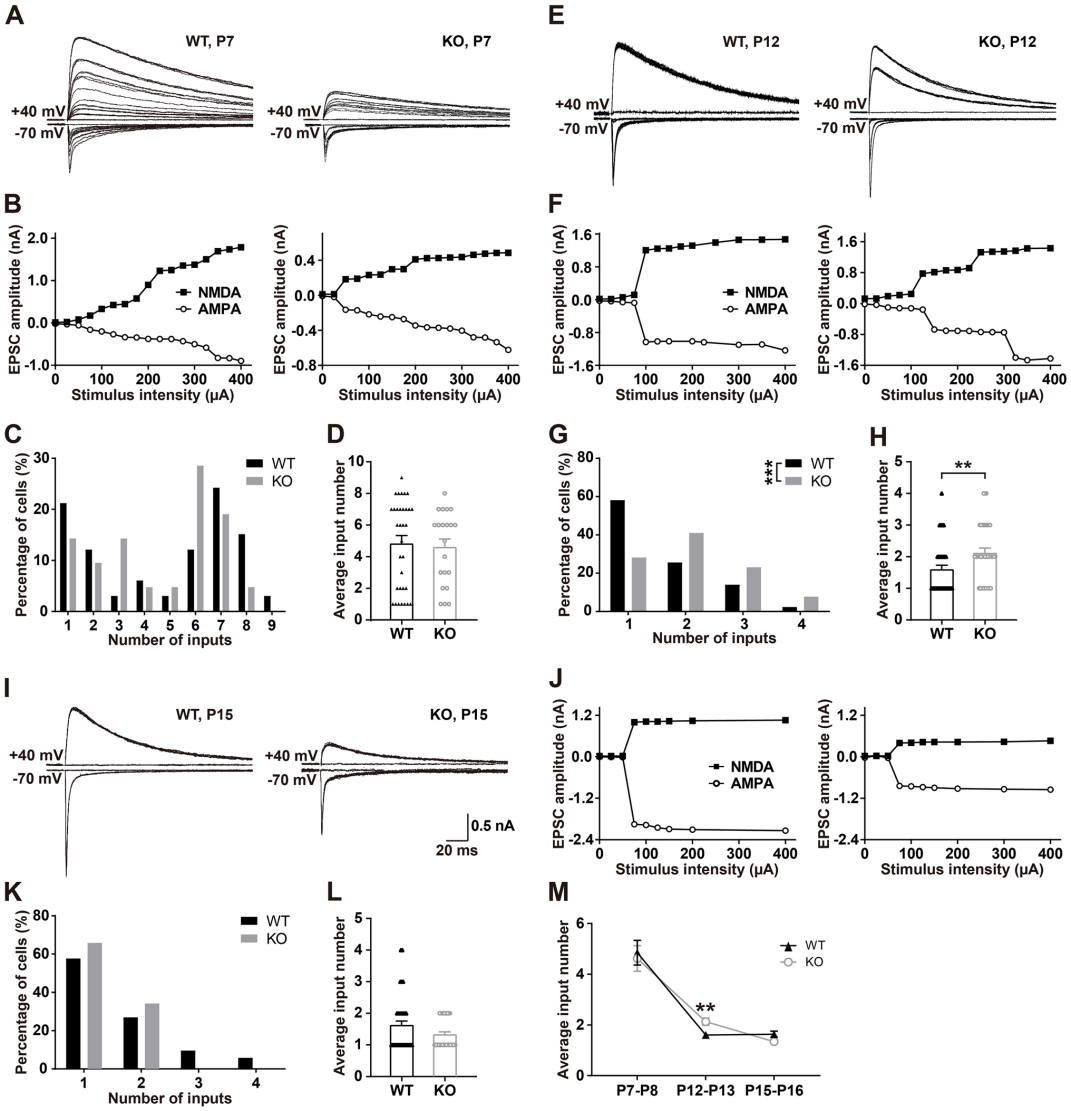
Synapse elimination is delayed in *Fmr1* KO mice. **A**, **E**, **I** Sample traces of EPSCs in response to stimuli over a range of intensities in VPm neurons at P7–8 (**A**), P12–13 (**E**), and P15–16 (**I**) in WT (left panels) and *Fmr1* KO mice (right panels). NMDAR-mediated EPSCs are recorded at +40 mV (upper traces), and AMPAR-mediated EPSCs are recorded at −70 mV (lower traces). The scale bars apply to **A**, **E**, and **I**. **B**, **F**, **J** Peak amplitude *vs* stimulus intensity for WT (left) and *Fmr1* KO (right) mice at P7–8 (**B**), P12–13 (**F**), and P15–16 (**J**). **C**, **G**, **K** Distribution of VPm neurons receiving different numbers of PrV axon inputs at P7–8 (**C**), P12–13 (**G**), and P15–16 (**K**) in WT and *Fmr1* KO mice. ***P <0.001, χ^2^ test. **D**, **H**, **L** Average number of inputs from PrV to each VPm neuron at P7–8 (WT, *n =* 33 from 5 mice; *Fmr1* KO, *n =* 21 from 6 mice, **D**), P12–13 (WT, *n =* 43 from 6 mice; *Fmr1* KO, *n =* 39 from 8 mice, **H**) and P15–16 (WT, *n =* 52 from 13 mice; *Fmr1* KO, *n =* 41 from 8 mice, **L**) in WT and *Fmr1* KO mice. **M** Changes in average input number during development in WT and *Fmr1* KO mice. **P <0.01, unpaired Student’s *t* test and Mann-Whitney test. Error bars indicate SEM

To investigate whether developmental synapse elimination is disrupted, we first examined the PrV-VPm projection using whole-cell patch clamp recording in acute brain slices from *Fmr1* KO or WT littermates of different ages. We found at P7, that the number of inputs received by each VPm neuron was comparable between the WT and *Fmr1* KO mice (*P* = 0.76, Fig. 1A–D). The mean number of PrV inputs received by each VPm neuron was 4.6 ± 0.50 in *Fmr1* KO mice and 4.8 ± 0.49 in WT littermates. However, a remarkable difference in the mean number of inputs received by each VPm neuron was noted at P12–13 (WT = 1.6 ± 0.13, *n =* 43 cells from 6 mice; *Fmr1* KO = 2.1 ± 0.14, *n =* 39 cells from 8 mice; *P* = 0.007, Fig. 1E–H). In WT mice, only 42% (18 of 43) of VPm relay neurons received multiple PrV inputs at this developmental stage, whereas most of these neurons (72%, 28 of 39) in *Fmr1* KO mice received multiple PrV inputs (Fig. 1G). These results indicate that the connectivity of the PrV-VPm projection had been impaired in P12–13 *Fmr1* KO mice. We considered that the redundant synaptic innervation of the VPm in *Fmr1* KO mice could be a result of a halt or delay in development. Therefore, we applied whole-cell patch clamp recording from VPm neurons at P15–16. At this later developmental stage, we found that the connectivity of the PrV-VPm projection in *Fmr1* KO mice had been restored to the WT level as the majority of VPm neurons from both *Fmr1* KO mice and WT littermates by then received only a single PrV input (WT = 1.6 ± 0.12, *n =* 52 cells from 13 mice; *Fmr1* KO = 1.3 ± 0.07, *n =* 41 cells from 8 mice; *P* = 0.20, Fig. 1I–L). These results suggested that the synaptic elimination deficit in *Fmr1* KO mice is due to a delay in, rather than a halting of, development (Fig. 1M).

### Pruning of Somatic Innervation is Disrupted in *Fmr1* KO Mice at P12–13

**Fig. 2:**
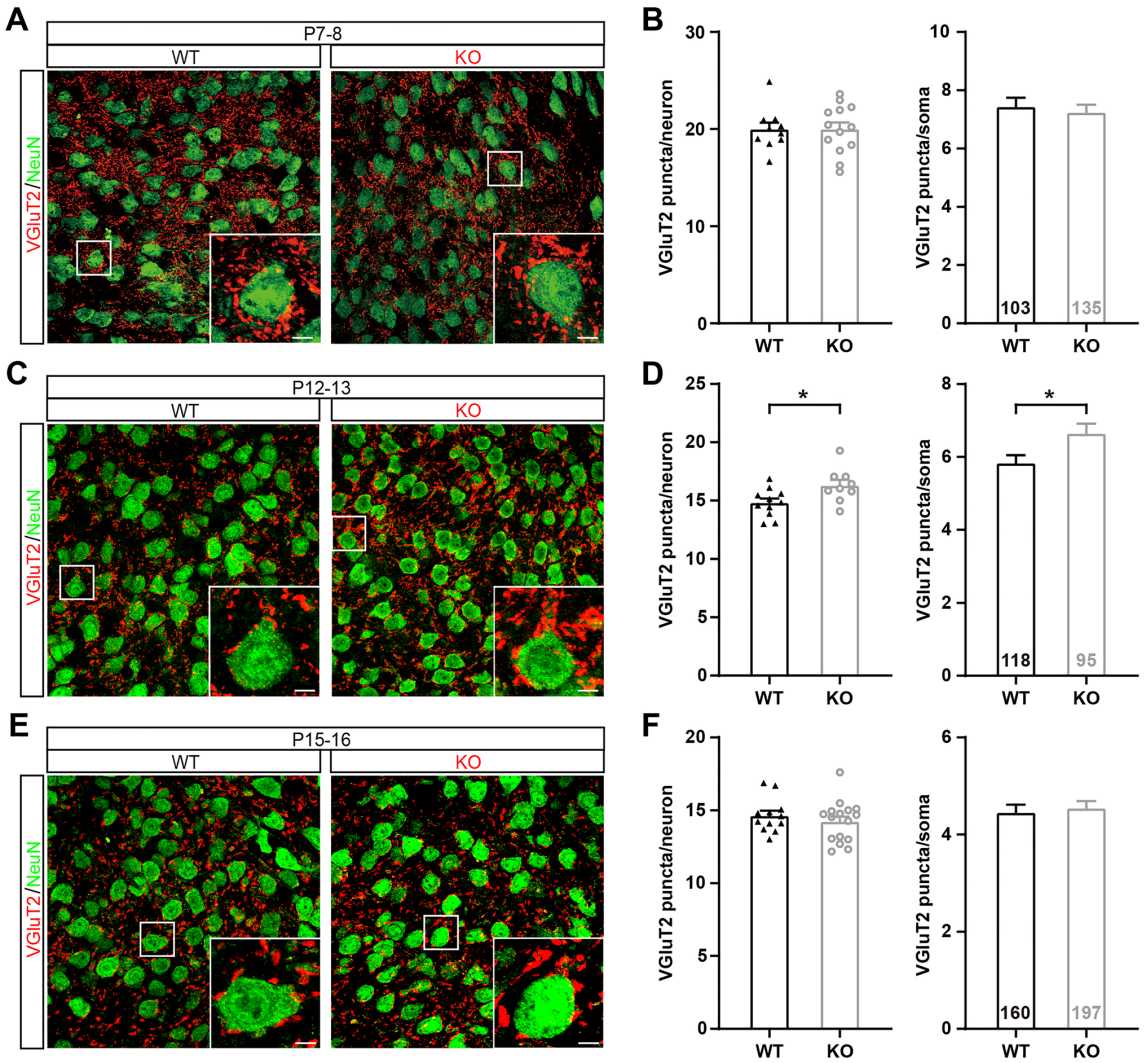
Pruning of somatic innervation is disrupted in *Fmr1* KO mice at P12–13. **A**, **C**, **E** Sample confocal images of VPm neurons and PrV axonal terminals in WT (left) and *Fmr1* KO (right) mice at P7–8 (**A**), P12–13 (**C**), and P15–16 (**E**). VPm neurons are visualized using the NeuN antibody (green). PrV axonal terminals are visualized using the VGluT2 antibody (red). Scale bars, 5 μm. **B**, **D**, **F** VGluT2 puncta/neuron (left) and VGluT2 puncta/soma (right) for WT and *Fmr1* KO mice at P7–8 (WT, *n =* 10 slices, *n =* 3 mice; *Fmr1* KO, *n =* 13 slices, *n =* 3 mice, **B**), P12–13 (WT, *n =* 11 slices, *n =* 3 mice; *Fmr1* KO, *n =* 9 slices, *n =* 3 mice, **D**), and P15–16 (WT, *n =* 12 slices, *n =* 3 mice; *Fmr1* KO, *n =* 16 slices, *n =* 4 mice, **F**). *P <0.05, unpaired Student’s *t* test and Mann-Whitney test. Error bars indicate SEM

VPm relay neurons receive two major excitatory inputs, one from layer VI of the cortex and the other from the PrV, that express vesicular glutamate transporters 1 and 2 (VGluT1 and VGluT2), respectively [22]. The number of inputs shown in the electrophysiological recording represents how many axonal projections are received by a given VPm neuron. Each PrV input forms multiple synaptic contacts with the soma of VPm neurons as revealed by VGluT2 staining [23, 24]. To further compare the number of synapses in the VPm between *Fmr1* KO and WT mice, we immunostained for VGluT2. We observed more VGluT2 puncta around the soma as well as increased the total number of puncta in *Fmr1* KO mice only at P12–13 (puncta/neuron: WT = 14.81 ± 0.36, *Fmr1* KO = 16.28 ± 0.49, *P* = 0.02; puncta/soma: WT = 5.83 ± 0.22, *Fmr1* KO = 6.64 ± 0.28, *P* = 0.02), but not at P7–8 (puncta/neuron: WT = 19.98 ± 0.68, *Fmr1* KO = 19.99 ± 0.70, *P* = 0.99; puncta/soma: WT = 7.43 ± 0.32, *Fmr1* KO = 7.24 ± 0.27, *P* = 0.57) or at P15–16 (puncta/neuron: WT = 14.63 ± 0.34, *Fmr1* KO = 14.23 ± 0.35, *P* = 0.43; puncta/soma: WT = 4.45 ± 0.17, *Fmr1* KO = 4.54 ± 0.15, *P* = 0.51; Fig. 2A–F). These results were consistent with the electrophysiological data that suggested a delay in synaptic elimination in *Fmr1* KO mice and indicated that the pruning of somatic innervation was deferred in *Fmr1* KO mice.

### Developmental Synapse Strengthening is Abnormal in *Fmr1* KO Mice

**Fig. 3:**
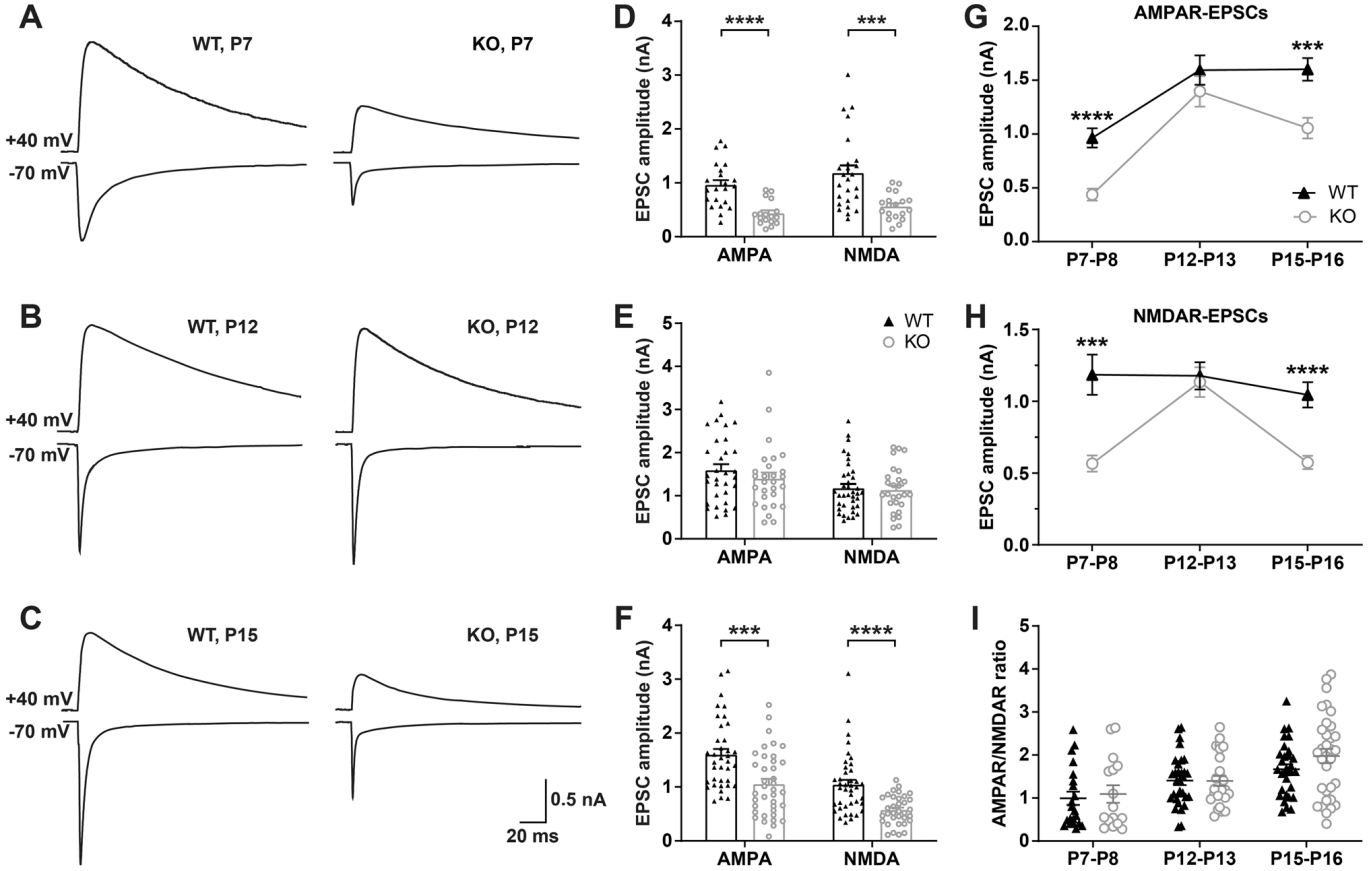
Synaptic strengthening is reduced in *Fmr1* KO mice at P7–8 and P15–16. **A–C** Sample traces of maximal evoked EPSCs from VPm neurons in WT (black) and *Fmr1* KO mice (red) at P7–8 (**A**), P12–13 (**B**), and P15–16 (**C**). The scale bar applies to all. **D–F** Peak amplitudes of evoked AMPAR-EPSCs and NMDAR-EPSCs in WT and *Fmr1* KO mice at P7–8 (WT, *n =* 5 mice; *Fmr1* KO, *n =* 5 mice, **D**), P12–13 (WT, *n =* 6 mice; *Fmr1* KO, *n =* 8 mice, **E**) or P15–16 (WT, *n =* 13 mice; *Fmr1* KO, *n =* 8 mice, **F**). **G**, **H** Changes in peak amplitude of AMPAR-EPSCs and NMDAR-EPSCs *vs* age in WT and *Fmr1* KO mice. **I** Changes in AMPAR/NMDAR ratio during development in WT and *Fmr1* KO mice. ***P <0.001, ****P <0.0001, unpaired Student’s t test and Mann-Whitney test. Error bars indicate SEM

Next, we compared the synapse function in *Fmr1* KO mice with WT littermate controls at different ages using whole-cell patch clamp recording from the VPm relay neurons. The maximal AMPAR-EPSCs and NMDAR-EPSCs of VPm neurons were evoked at the same intensity of stimuli applied to the medial lemniscus when the membrane potential was held at − 70 mv and +40 mv, respectively. The AMPAR-mediated component of EPSCs was determined by measuring the peak amplitude of the EPSC at − 70 mV. At +40 mV, the AMPAR-EPSC was very small, due to strong inward rectification, and decayed rapidly, suggesting that the VPm relay synapse contains very few GluR2 subunits [20]. Thus, the peak amplitude of the EPSC at +40 mV is suggested to be almost entirely mediated by NMDARs. Our data showed that at P7–8, the synaptic properties, including AMPAR-EPSCs (*Fmr1* KO, 437.6 ± 54.74 pA; WT, 963.5 ± 89.03 pA, *P* <0.0001), and NMDAR-EPSCs (*Fmr1* KO, 565.6 ± 56.41 pA; WT, 1185 ± 139.8 pA, *P* = 0.0006) were significantly reduced in *Fmr1* KO mice as compared to the corresponding values in WT littermates (Fig. 3A, D).

In contrast, the synaptic function recorded at VPm relay synapses was comparable between *Fmr1* KO and WT control mice at P12–13 (AMPAR-EPSCs: *Fmr1* KO = 1397 ± 141.9 pA, WT = 1593 ± 135.9 pA, *P* = 0.32; NMDAR-EPSCs: *Fmr1* KO = 1134 ± 103.2 pA, WT = 1177 ± 94.6 pA, *P* = 0.76) (Fig. 3B, E). However, when comparing the synaptic properties at P15–16, *Fmr1* KO mice again showed a remarkable difference from WT mice, showing reduction in AMPAR-EPSCs (*Fmr1* KO, 1055 ± 96.6 pA; WT, 1600 ± 105.1 pA; *P* = 0.0003), and NMDAR-EPSCs (*Fmr1* KO, 573.4 ± 46.2 pA; WT, 1045 ± 88.2 pA; *P* <0.0001, Fig. 3C, F). Although no significant difference was found in the AMPAR/NMDAR ratio between *Fmr1* KO and WT control mice during early development, our results suggest that there was a temporary recovery of synaptic function in the VPm at P12–13, but that synaptic strengthening was reduced in *Fmr1* KO mice at both P7–8 and P15–16 (Fig. 3 G–I).

### The Density and Size of Synaptic Vesicles are Abnormal in *Fmr1* KO Mice During Development

**Fig. 4:**
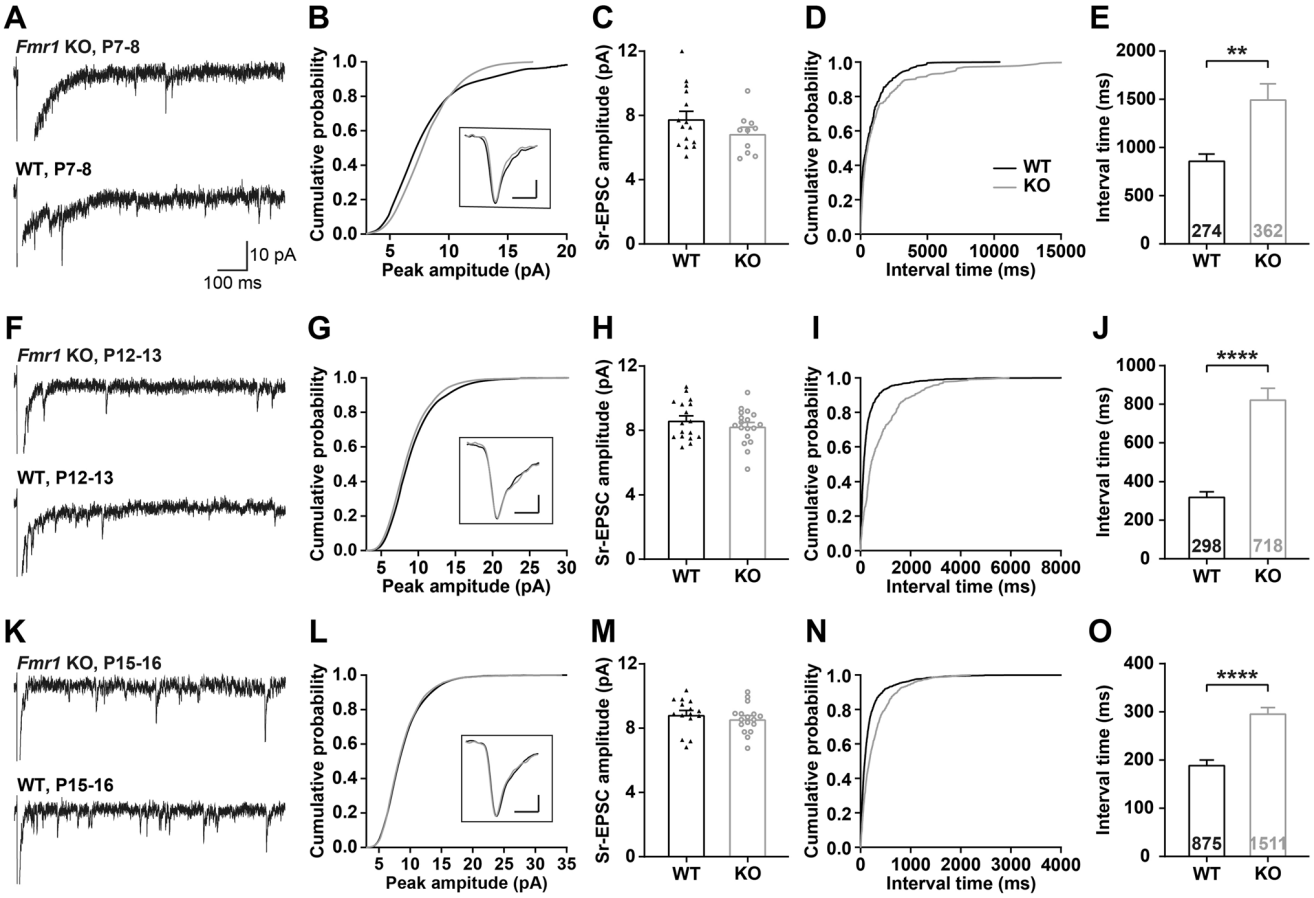
Evoked quantal events of VPm neurons in *Fmr1* KO and WT mice. **A**, **F**, **K** Sample traces of evoked quantal EPSCs in *Fmr1* KO (red) and WT (black) mice at P7–8 (**A**), P12–13 (**F**) and P15–16 (**K**). The scale bar applies to all. **B**, **G**, **L** Cumulative distribution of the peak amplitude for WT (black) and *Fmr1* KO (red) mice at P7–8 (**B**), P12–13 (**G**), and P15–16 (**L**). Insets: averaged Sr-EPSCs from WT (black) and *Fmr1* KO (red) mice. The scale bars in the inset represent 2 pA and 2 ms. **C**, **H**, **M** Mean peak amplitudes of Sr-EPSCs recorded from WT and *Fmr1* KO mice at P7–8 (WT, *n =* 15 cells, *n =* 3 mice; *Fmr1* KO, *n =* 9 cells, *n =* 3 mice, **C**), P12–13 (WT, *n =* 17 cells, *n =* 3 mice; *Fmr1* KO, *n =* 18 cells, *n =* 3 mice, **H**), and P15–16 (WT, *n =* 15 cells, *n =* 3 mice; *Fmr1* KO, *n =* 17 cells, *n =* 3 mice, **M**), *P* >0.05, unpaired Student’s *t* test). **D**, **I**, **N** Cumulative distribution of the interval time for WT (black) and *Fmr1* KO (red) mice at P7–8 (**D**), P12–13 (**I**), and P15–16 (**N**). **E**, **J**, **O** Mean interval time of Sr-EPSCs recorded from WT and *Fmr1* KO mice at P7–8, P12–13, and P15–16. **P <0.005, ****P <0.0001, Mann-Whitney test. Error bars indicate SEM

**Fig. 5:**
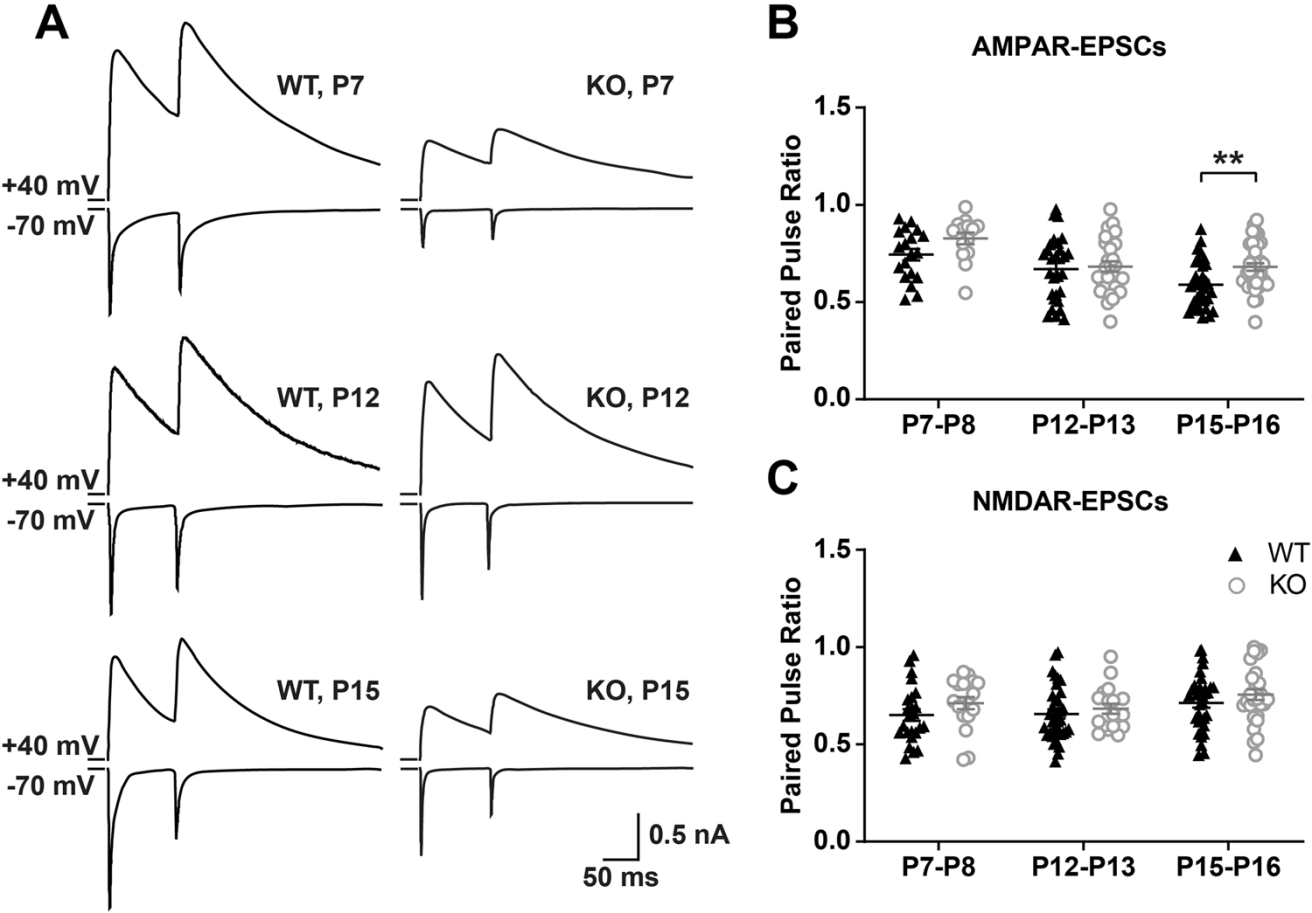
Release probability is largely unaffected in *Fmr1* KO mice during development. **A** Sample traces of paired-pulse responses recorded at +40 mV and −70 mV from VPm neurons in WT (left) and *Fmr1* KO (right) mice at P7–8 (upper), P12–13 (middle), and P15–16 (lower). **B**, **C** Paired pulse ratio of AMPAR-EPSCs (upper) and NMDAR-EPSCs (lower) *vs* age for WT and *Fmr1* KO mice (P7–8, *n =* 5 mice in WT and 5 in *Fmr1* KO; P12–13, *n =* 6 mice in WT and 8 in *Fmr1* KO; P15–16, *n =* 13 mice in WT and 8 in *Fmr1* KO). The inter-pulse interval is 100 ms. **P < 0.01, unpaired Student’s t test and Mann-Whitney test. Error bars indicate SEM

It is important to note that the VGluT2 immunostaining results could not explain the developmental deficits of synaptic function in the VPm. To further explore the causes of impairment of synaptic function in *Fmr1* KO mice, we next recorded evoked quantal EPSCs at –70 mV in WT and *Fmr1* KO mice at P7–8, P12–13, and P15–16. Using Sr^2+^ to replace Ca^2+^ in the ACSF reduced the peak amplitude of EPSCs at –70 mV and caused a large number of asynchronous quantal EPSCs (Sr–EPSCs; Fig. 4A, F, K). The mean amplitude of Sr-EPSCs in VPm neurons of *Fmr1* KO mice was similar to that in neurons of WT mice during P7–8, P12–13, and P15–16 (Fig. 4B, C, G, H, L, M). In contrast, the interval between each quantal event was significantly longer in *Fmr1* KO mice than in WT mice (P7–8: WT = 868.1 ± 63.67 ms, n of 15 cells from 3 mice, *Fmr1* KO = 1502 ± 158.5 ms, n of 9 cells from 3 mice, *P* = 0.0010, Fig. 4D, E; P12–13: WT = 323.6 ± 24.56 ms, n of 17 cells from 3 mice, *Fmr1* KO = 826.3 ± 56.76 ms, n of 18 cells from 3 mice, *P* <0.0001, Fig. 4I, J; P15–16: WT = 190.4 ± 9.70 ms, n of 15 cells from 3 mice, *Fmr1* KO = 297 ± 11.68 ms, n of 17 cells from 3 mice, *P* <0.0001, Fig. 4N, O). These results suggest that the impairment of synaptic function in the VPm of *Fmr1* KO mice is not attributable to any postsynaptic mechanism. We next tested the presynaptic vesicle release probability (PPR) in the PrV-VPm synapses. We found that, except for the PPR of AMPAR-EPSCs at P15–16, there was no significant difference in the PPR for *Fmr1* KO mice compared to WT mice at different ages (Fig. 5).

**Fig. 6:**
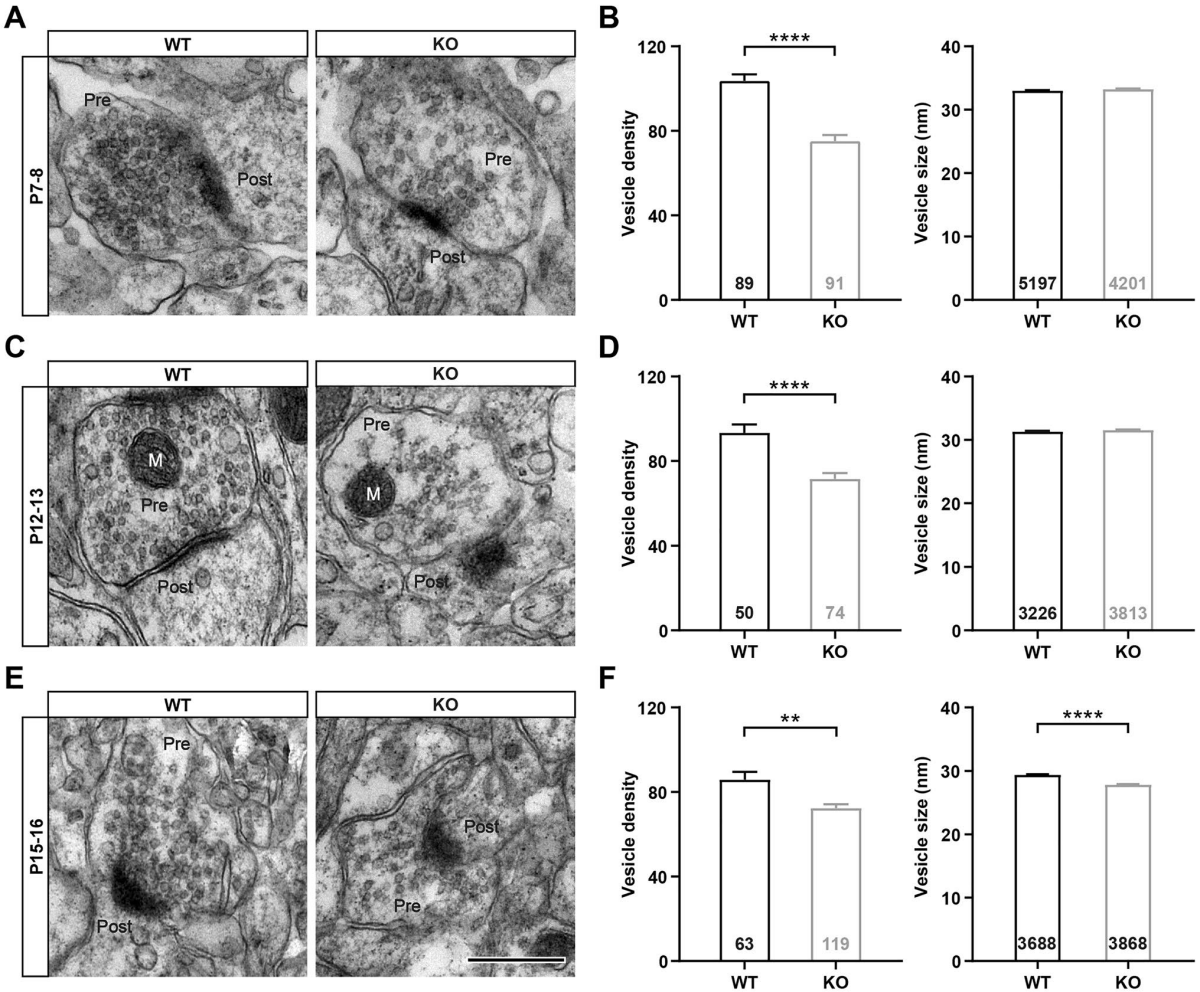
The density and size of synaptic vesicles are abnormal in *Fmr1* KO mice during development. **A**, **C**, **E** Representative TEM images of synapses on VPm neurons from WT (left) and *Fmr1* KO (right) mice at P7–8 (**A**), P12–13 (**C**), and P15–16 (**E**). Pre, pre-synaptic area; Post, post-synaptic area; M, mitochondria. Scale bar, 500 nm. **B**, **D**, **F** Comparisons of synaptic vesicle density (left, vesicle number per synapse area in μm^2^) and each vesicle size (right) in WT and *Fmr1* KO mice at P7–8 (WT, *n =* 89 synapses and 5,197 vesicles from 3 mice; *Fmr1* KO, *n =* 91 synapses and 4,201 vesicles from 4 mice, **B**), P12–13 (WT, *n =* 50 synapses and 3,226 vesicles from 3 mice; *Fmr1* KO, *n =* 74 synapses and 3,813 vesicles from 3 mice, **D**), and P15–16 (WT, *n =* 63 synapses and 3,688 vesicles from 3 mice; *Fmr1* KO, *n =* 119 synapses and 3868 vesicles from 3 mice, **F**). **P <0.01, ****P <0.0001, unpaired Student’s *t* test and Mann-Whitney test. Error bars indicate SEM

**Fig. 7:**
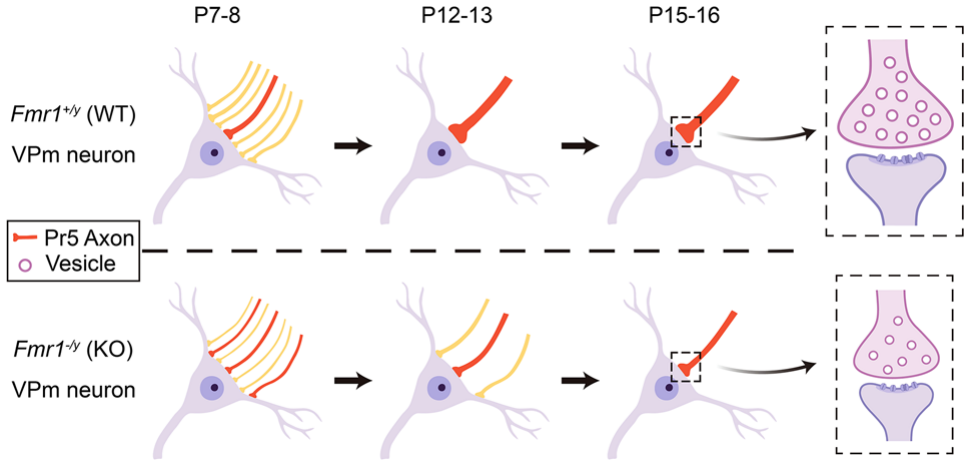
Schematic of the refinement process of thalamic sensory synapses during early development (postnatal days 7–16) in *Fmr1* WT (upper) and KO (lower) mice. *Fmr1* KO mice display abnormal synaptic refinement, including delay of synaptic elimination and decline of individual synaptic function. The proposed presynaptic mechanism of deformities in synaptic vesicle number and size may be responsible for the deficits in developmental synaptic strengthening (dashed box)

We then investigated the size and number of synaptic vesicles in PrV terminals that contact VPm neurons. The VPm regions of *Fmr1* KO and WT mice at the ages of P7–8, P12–13, and P15–16 were sampled and imaged by electron microscopy. Our data revealed that the vesicle density in the presynaptic terminals of *Fmr1* KO mice was significantly lower than that of WT mice at P7–8, P12–13, and P15–16. However, the vesicle size of *Fmr1* KO mice was decreased significantly compared to WT mice only at P15–16 (WT = 29.37 ± 0.12, *n =* 3688 vesicles; *Fmr1* KO = 27.83 ± 0.10, *n =* 3868; *P* <0.0001), with no significant differences at P7–8 (WT = 33.02 ± 0.11, *n =* 5197; *Fmr1* KO = 33.25 ± 0.11, *n =* 4201, *P* = 0.15) or P12–13 (WT = 31.33 ± 0.12, *n =* 3226; *Fmr1* KO = 31.54 ± 0.12, n= 3813, *P* = 0.23; Fig. 6A–F). These results suggest that the size and number of presynaptic vesicles in the VPm of *Fmr1* KO mice are abnormal during early development, while the probability of vesicle release is not significantly altered. We considered that such a reduction in the number of synaptic vesicles in PrV terminals might be the reason for the significantly impaired function of individual synapses in the VPm of the *Fmr1* KO mice compared to WT mice (Fig. 7).

## Discussion

In this study, we found abnormalities in the synaptic refinement process of thalamic sensory synapses during early development in *Fmr1* KO mice, including delay of elimination and decline of synaptic function, indicating possible anomalies in the somatosensory pathway. The early development of the somatosensory pathway not only plays an important role in the tactile information process but also serves as a fundamental contributor to the maturation of the oxytocinergic signaling pathway and the correct establishment of social communication in many mammalian species including humans and rodents. It is well-accepted that whisker deprivation in early life reduces the brain’s oxytocin (OXT) level, a hormone that is vital for social behaviors [25]. In addition, a reduction of plasma and cerebral OXT levels has been reported in *Fmr1* KO mice [11]. This coincidence suggests that the developmental deficit of the somatosensory system may contribute as a potential mechanism for the reduced OXT and impaired social function in *Fmr1* KO mice.

Synaptic refinement in the central nervous system is a developmental process necessary for the establishment of proper connectivity and function [26]. In particular, a number of studies have demonstrated that abnormal synaptic pruning underlies a variety of neurodevelopmental disorders including ASDs [27–31]. The nature of the developmental characteristics of the PrV-VPm projection provides a good opportunity to investigate the synaptic connection structure and synaptic function.

In a previous study, we found that the developmental elimination of the PrV-VPm relay synapse is accomplished at ~P16–17 in C57BL/6J mice. Prior to this, at the approximate age of P12–13, the majority of VPm neurons have been noted to still receive 2 or 3 PrV innervations [20]. However, in the current study with the FVB genetic background mouse strain, developmental synapse elimination was completed at P12–13. These results suggest that different mouse strains may have distinct developmental features. This issue requires consideration for future studies.

Our results, including electrophysiological recording and VGluT2 immunostaining data, suggest a delay in developmental synapse elimination at PrV-VPm synapses in *Fmr1* KO mice. However, the strength of excitatory synapses formed by PrV projections onto VPm neurons was significantly weaker in *Fmr1* KO mice than in WT littermates during early development. Although at P12–13, the peak amplitude of AMPAR-EPSCs and NMDAR-EPSCs were comparable between *Fmr1* KO mice and control mice, given there are so many more somatic synapses in *Fmr1* KO mice than the WT at this age, the function per individual synapse can still be considered to be much lower. These results suggest an abnormal strengthening of thalamic somatosensory synapses in *Fmr1* KO mice at various early developmental ages. This phenomenon is in line with the finding that a reduced Sr-EPSC frequency, but not amplitude at different time points in *Fmr1* KO mice when compared to those in WT mice, suggesting the impaired function of VPm relay synapses is attributable to a presynaptic mechanism. Correspondingly, our electron microscopy data also consistently showed that vesicle density was significantly decreased in *Fmr1* KO mice at the various early developmental ages investigated. This suggests a presynaptic mechanism contributing to the deficits in developmental synaptic strengthening in *Fmr1* KO mice. However, we cannot exclude the role of post-synaptic FMRP participating in synaptic function or synaptic elimination in *Fmr1* KO mice. To clarify these caveats, cell-type, and region-specific KO experiments are necessary, and need to be carried out in the future. Considering that deficits in synaptic function occur earlier than those in synaptic pruning, and the process of developmental synaptic elimination is neural activity-dependent, the delayed synaptic pruning found in the current study could potentially be caused by insufficient neural activity produced either in the PrV of the brainstem or *via* the vibrissae in *Fmr1* KO mice. Alternatively, glial cells including microglia and astrocytes could be involved, since they also play important roles in synaptic pruning during postnatal development [32–36] and in FXS disorder where the absence of FMRP leads to impaired phagocytosis of postsynaptic structures mediated by microglia in the hippocampus [37]. This latter study also revealed significantly reduced levels of synaptic gaps contacted by astrocytes in the hippocampus of *Fmr1* KO mice.

Since the thalamus is considered to be a subcortical processing center for the relay of sensory information from the periphery to the cortex [38], such a developmental impairment of the VPm relay synapse may contribute to abnormalities in the sensory processing of *Fmr1* KO mice. Furthermore, new evidence suggests that the thalamus continues to contribute to the processing of information within cortical hierarchies. For example, recent studies have demonstrated that, in addition to the critical role of filtering and coordinating sensory information, the thalamus is also involved in modulating sensory information processing by maintaining and regulating functional connections within cortical areas through thalamocortical circuits [38]. In the somatosensory pathway, perhaps the best example of a trans-thalamic pathway is the projection in the mouse from layer V of the primary somatosensory cortex to the higher-order posterior medial nucleus and then onto the secondary somatosensory cortex [39, 40]. The complexity of such thalamocortical circuits and the fact that the developmental deficit of synaptic refinement in *Fmr1* KO mice strongly suggest that the thalamic contribution should be considered in the study of somatosensory over-reactivity in FXS.
